# Rescue Saline Washout for Popliteal Sciatic Block

**DOI:** 10.7759/cureus.103784

**Published:** 2026-02-17

**Authors:** Viet Scott C Nguyen, Joseph E Villaluz, William Mitchell

**Affiliations:** 1 Anesthesiology, University of California, Irvine (UCI) Medical Center, Orange, USA; 2 Anesthesiology and Perioperative Medicine, University of California, Irvine (UCI) Health, Orange, USA; 3 Anesthesia and Critical Care, University of California, Irvine (UCI), Irvine, USA

**Keywords:** nerve block, peripheral nerve block analgesia, popliteal sciatic, regional anesthesiology, saline lavage

## Abstract

Rescue saline washouts have been reported to reverse the effects of local anesthetic blockade in interscalene blocks and epidurals; however, evidence for their use in lower-extremity blocks is limited. This case report discusses a scenario in which a 73-year-old, 62.4 kg, female patient received an ultrasound-guided preoperative popliteal sciatic nerve block for lower extremity surgery. Subsequently, due to unforeseen circumstances, the case was cancelled, and thus the effects of the block were no longer desired. The patient wished to return home but required motor function at that time. To preserve motor function, a normal saline washout was performed at the same popliteal sciatic level under ultrasound guidance (Sonosite, Bothell, Washington). Administration of normal saline using an echogenic needle (Pajunk, Geisingen, Germany) was associated with some return of sensory function as early as 30 minutes after rescue and of motor function at three hours post-washout. This case suggests the plausibility of using a known technique, such as saline washout, to reverse the effects of nerve blockades in the setting of popliteal sciatic blocks.

## Introduction

Peripheral nerve blocks are a common and highly effective method for mitigating perioperative pain, particularly in extremity surgeries. By using local anesthetics, which block intracellular sodium channels, both sensory and motor blockade can be achieved for surgical anesthesia and analgesia. In addition to providing sensory blockade to reduce pain, however, some peripheral nerve blocks can cause undesirable effects, such as motor blockade or phrenic nerve palsy with interscalene blockades.

Previous literature has described successful alleviation of respiratory distress in phrenic nerve palsy secondary to interscalene blocks with rescue saline washouts [1-5]; however, no studies have reported the use of rescue saline washouts to relieve motor blockade in lower-extremity blocks. Although the mechanism of saline washouts is not fully elucidated, it may reverse the effects of local anesthetic blockade by dilution, altering pH, or changing sodium concentration [6].

This case report presents a successful rescue saline washout for a popliteal sciatic block, with recovery of motor and sensory function, in a patient who required an expedited return to ambulation.

## Case presentation

A 73-year-old, 62.4 kg, female patient with a past medical history of insulin-dependent type 1 diabetes with known peripheral neuropathy, contracture of the right Achilles tendon, and cavus deformity of the right foot, presented for an elective right ankle arthrodesis with allograft and Achilles tendon lengthening. Of note, the patient had preexisting numbness on the plantar aspect of her right foot; otherwise, gross sensation and motor function were intact.

The patient was scheduled for the first case of the day in the operating room. Anesthetic planning included ultrasound-guided (Sonosite, Bothel, Washington) preoperative nerve block for postoperative pain control in addition to general anesthesia. The regional anesthesia team placed a single-shot adductor canal (10 mL of ropivacaine 0.5%) and sciatic popliteal fossa block (20 mL of ropivacaine 0.5%) at 0630 using an echogenic needle (Pajunk, Geisingen, Germany). Ropivacaine 0.5% was used to maximize the duration and effectiveness of the postoperative block.

The block was deemed successful, with the expected distribution of sensation and motor function observed in both blocks. Due to bed capacity constraints, the patient’s elective case was cancelled, and the patient no longer qualified for the original plan of 24-hour hospital observation. The patient’s surgery was postponed by two days; however, she was discharged the same day and returned two days later for the rescheduled procedure. This posed a significant challenge, considering the patient lived in a multiple-story home alone with minimal support and an effective motor nerve block that would impede safe ambulation and activities of daily living.

**Figure 1:**
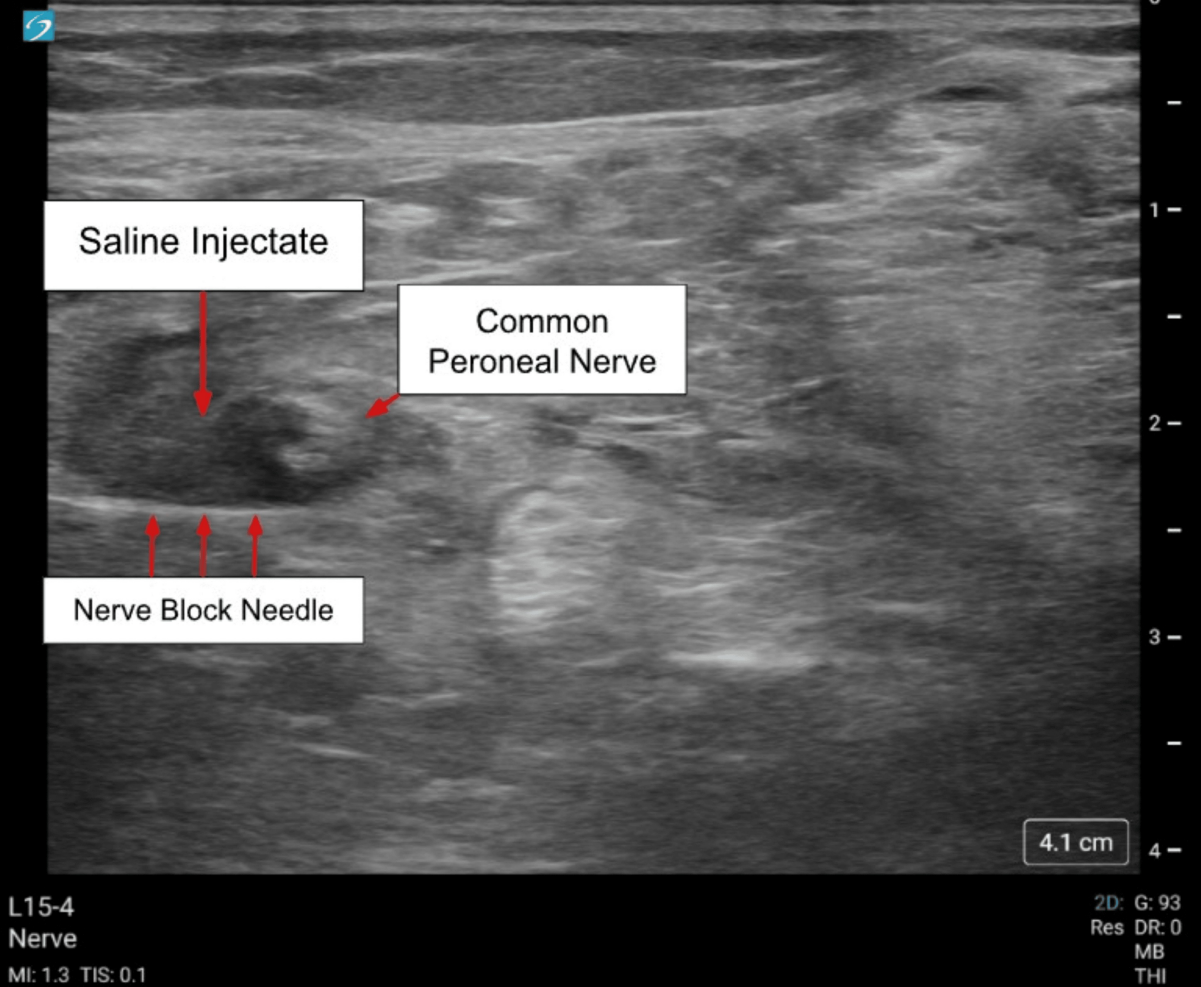
0.9% normal saline surrounding the common peroneal nerve (left side of screen indicates lateral). Sonosite ultrasound was utilized with L15-4 MHz linear transducer and the Pajunk Sonoplex II needle.

**Figure 2:**
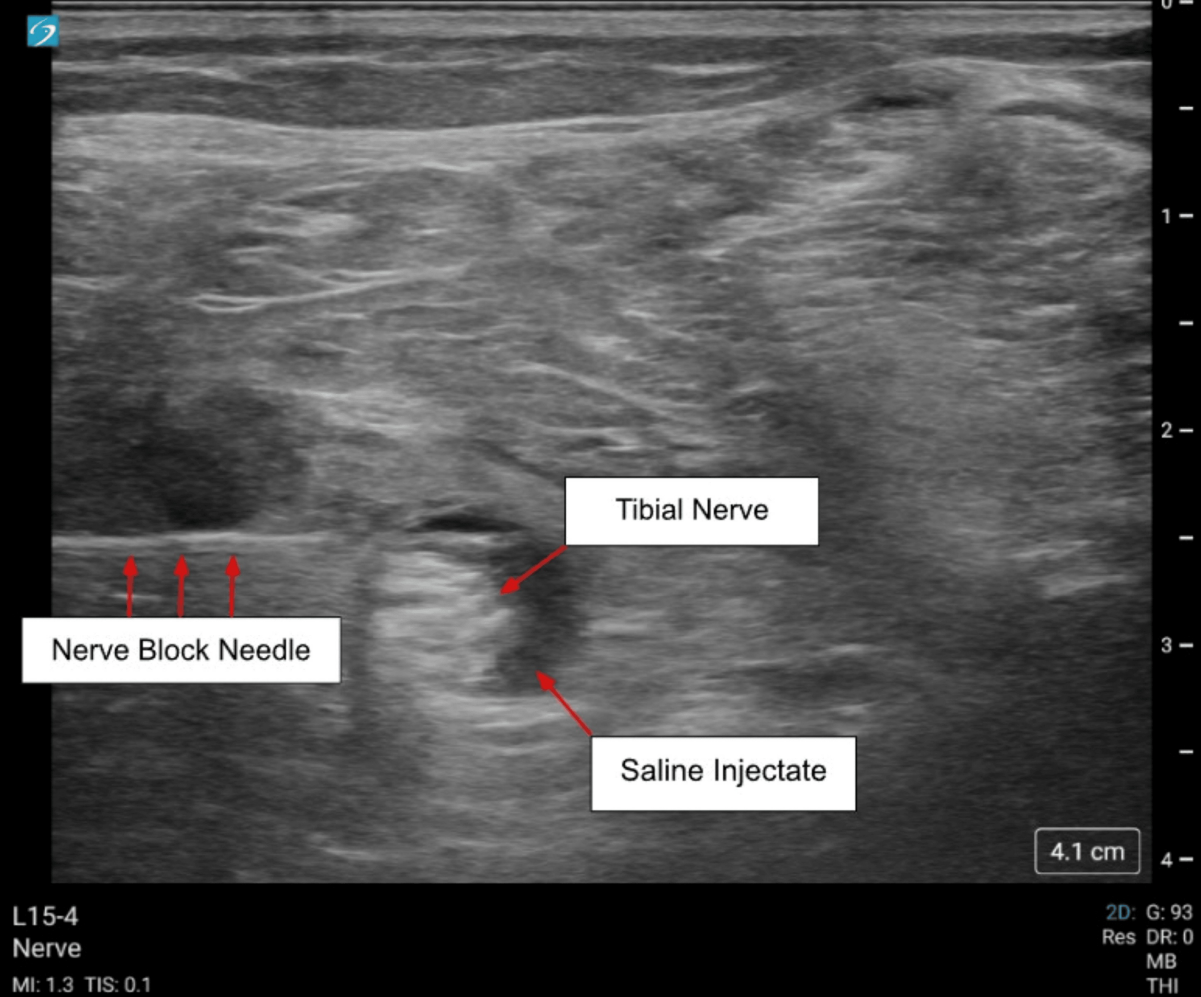
0.9% normal saline surrounding the tibial nerve (left side of screen indicates lateral). Sonosite ultrasound was utilized with the L15-4 MHz Linear Transducer and Pajunk Sonoplex II needle.

Given the circumstances, a rescue saline washout was offered to the patient, which she accepted. Prior to performing the rescue washout, the patient continued to exhibit appropriate sensory and motor blockade consistent with the aforementioned blocks. Given the primarily sensory nature of the adductor canal block, reversal of the sciatic nerve block took precedence. As a salvage technique with hopes for return of motor function, a popliteal sciatic saline washout was performed under ultrasound guidance about three hours after the initial block (at 1000), administering a total of 25 mL of 0.9% normal saline to approximate a similar volume as the initial block without causing discomfort (Figures 1-2).

Within the first 30 minutes of the rescue saline washout, the patient reported regaining some sensation. Physical therapy was then consulted to evaluate the patient’s ability to ambulate. On evaluation by anesthesiology one hour after the washout, she noted some dorsiflexion and plantarflexion at 1+/4 strength. At six and a half hours after the initial block (three hours after the rescue washout), the physical therapists noted that the patient had “light and deep touch to the lower extremity,” was able to perform the “sit and stand” maneuvers with a four-wheel walker, and was also able to ambulate 200 feet with the walker. Given the short interval between the initial block and the recovery of modest sensation and motor function, it was highly plausible that the rescue saline washout was effective. The patient was discharged the same day and returned for surgery two days later without incident.

## Discussion

Local anesthetics block the propagation of pain signals by inhibiting voltage-gated sodium channels, thereby interrupting action potentials. Termination of action for local anesthetics is usually determined by drug metabolism, but the mechanism of saline washouts in discontinuing the effects of a local anesthetic block remains incompletely understood. In a case report, Meulemans and Gerritse performed a single-shot saline rescue injection to manage respiratory difficulty after an interscalene block. They hypothesized that dilution of the local anesthetic, pH changes, or changes in sodium concentration were responsible for the effectiveness of the saline washout [6].

**Figure 3:**
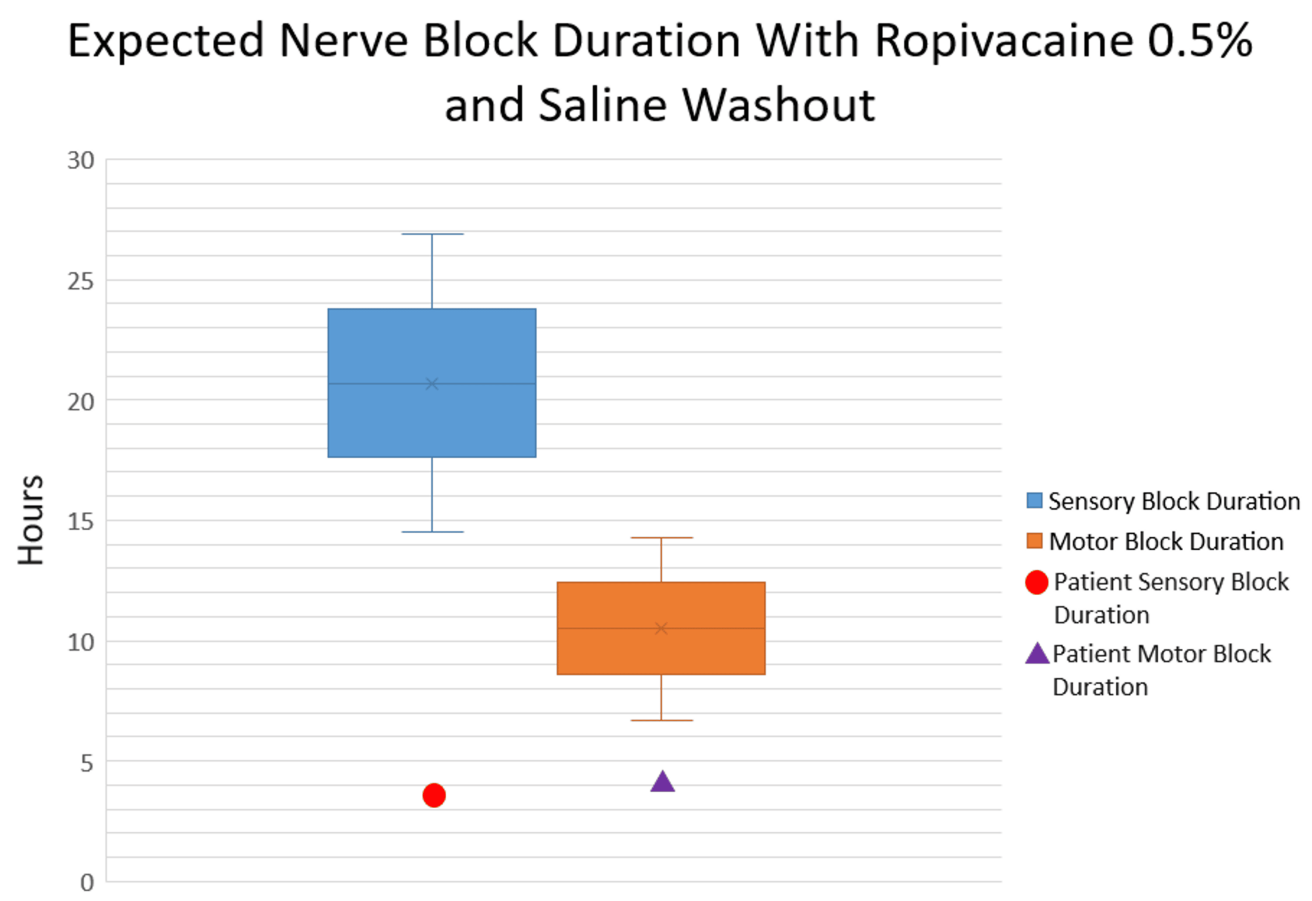
Expected sensory block duration range per Fernandez-Guisasola et al. (blue, 20.7 + 6.2 hours) and motor block duration range per Casati et al. (orange, 10.5 + 3.8 hours). The patient’s initial sensory recovery time (red circle, 3.5 hours) and initial motor recovery time (purple triangle, 4 hours) are depicted above. [7,8]

Another point to consider is a comparison between the expected durations of sensory and motor block and those observed in this patient. In a study by Fernandez-Guisasola and colleagues comparing 0.5% ropivacaine and 1% mepivacaine for popliteal sciatic blocks, sensory blockade lasted about 20.7 + 6.2 hours [7]. Regarding the duration of motor blockade, Casati and colleagues reported that sciatic blockade with 0.5% ropivacaine lasted approximately 10.5 ± 3.8 hours [8]. Using these as references, this patient appeared to have regained modest sensory and motor function faster than expected at 3.5 and 4 hours after the initial block, respectively (Figure 3).

The expected pattern of neurological recovery after peripheral nerve blockade is motor first, followed by sensory recovery, owing to the nature of the nerves themselves. Sensory fibers (C fibers) are smaller than motor fibers (A-alpha), and as such would be the first to be blocked and last to recover. For this patient, however, she reported that sensation returned before meaningful motor restoration. This pattern is atypical, but the authors have not elucidated the possible mechanism.

In general, the application of a rescue saline washout has not been widely described in the current literature, let alone widely used in clinical practice. Most of the current literature on the reversal of peripheral nerve blockades derives from case reports aimed at reversing phrenic nerve palsy [1-5]. In addition to case reports, Gerber et al. performed a randomized controlled trial in which 10 mL of normal saline was injected through an interscalene catheter to reverse phrenic nerve blockade after the initial single-shot injection [9]. Although the washout did not reduce phrenic blockade or restore diaphragmatic function, it did achieve a statistically significant reduction in the progression to full diaphragmatic paralysis. Of note, analgesia was preserved in this study.

Although the literature described above suggests the possibility of reversing the adverse effects of interscalene blocks (particularly phrenic nerve palsy), a study by Byrne et al. [10] investigating saline washout after supraclavicular and infraclavicular blocks did not show faster resolution of motor and sensory blockade than in the control group. This suggests that the effectiveness of possible block reversal depends on the type of block and the type of effect the clinician seeks to reverse. That said, further clinical trials are needed to assess the effectiveness of saline rescues across different types of blocks.

One potential factor affecting the effectiveness of a saline washout is the initial local anesthetic concentration used for the block. As discussed above, sensory fibers require less local anesthetic to block because of their smaller diameter. In a noninferiority study by Wu et al. comparing 0.25% and 0.375% ropivacaine for popliteal sciatic blocks, 0.25% ropivacaine had a lower incidence of motor blockade but maintained sensory blockade and patient satisfaction [11]. Considering these findings, using a lower concentration during the initial block could facilitate a more rapid return of motor function while maintaining sensory blockade for pain relief. That said, this idea requires further trials to determine its validity.

The clinical scenario described in this case report is significant because it is among the first in the current literature to use a rescue saline washout for a lower-extremity block, and it may allow nerve blockade reversal in a “no longer needed” scenario. The fact that the effects of the popliteal sciatic block were sufficiently reduced to permit ambulation with a walker and same-day discharge within hours is a promising sign that this may be a viable method for block reversal or at least warrants consideration in such circumstances. That said, further trials are needed to confirm the viability of rescue saline washouts for lower-extremity peripheral nerve blocks.

## Conclusions

In cases of a block that is “no longer needed” due to unforeseen circumstances, a rescue saline washout for a popliteal sciatic nerve block may provide a modest return of sensory and motor function. Further studies are needed before rescue washouts can be established as the standard of care.
